# Auto-Plastic Intra-Medullary Bone Pegging as a Method of Operative Treatment for Fractures

**Published:** 1914-06

**Authors:** C. Ferrier Walters

**Affiliations:** Assistant Surgeon, Bristol Royal Infirmary


### AUTO-PLASTIC INTRA-MEDULLARY BONE PEGGING AS A METHOD OF OPERATIVE TREATMENT FOR FRACTURES.

BY

C. Ferrier Walters, F.R.C.S.,
*Assistant Surgeon, Bristol Royal Infirmary.*

---

Methods of operative fixation of fractures have for some years engrossed the minds of surgeons, particularly in the search for a perfect fixation material, that is to say, one absorbable and non-irritant, causing neither delay nor excess in the formation of callus, but all materials so far utilised have failed in one or other respect.

With the perfect fixateur in view and its necessary adjuncts, absence of irritation and complete absorbability, it occurred to me that auto-plastic intra-medullary bone pegging might offer a solution of the difficulties, for the following reasons: That Macewen's recent work on callus formation has proved that it is chiefly derived from the bone itself, growing from every surface in the most suitable pabulum, namely blood-clot at the site of fracture; that one has on several occasions seen compact bone exposed in a compound fracture and apparently necrotic, revitalised by vascular permeation throughout its whole extent; that in the comminuted simple fracture, which almost invariably unites without undue delay, pieces of compact bone must sometimes be entirely separated and yet retain their vitality, or are absorbed without detriment to union, for how often does one in these cases see necrosis or failure in union occur; that the transplantation of bone is often successful; that the implantation of living bone into the medullary canal at the site of fracture should have no greater dangers attached to it than the introduction there of ivory pegs; that internal callus is formed and re-absorbed at the site of fracture, and it therefore would be probable that living bone as an intra-medullary peg might retain its vitality, or, like internal callus, be absorbed without causing delay in union.

Before attempting this method I found no record in the available literature of the introduction of living bone into the medullary canal for the fixation of fractures.

After my first three cases Greiffenhagen[1] reported two cases of pseudarthrosis which he had dealt with in 1911 by auto-plastic intra-medullary bone pegging, both of which failed.

In January of this year Albee,[2] of New York, reported a case of fracture of the neck of the femur in which he utilised, with success, a living bone peg from the patient's own fibula for fixation of the fracture.

Mr. Groves, in his excellent exhibition of the methods of operative fixation of fracture in March of this year, showed a skiagram which indicated the successful result of an intra-medullary bone peg for fractured tibia obtained by Professor Marnoch, of Aberdeen.

No doubt similar records have escaped my notice.

Four opportunities occurred to me in August, September and October last to judge the effect of auto-plastic intra-medullary bone pegging as a fixateur for fractures requiring operative treatment.

The first case was that of a somewhat delicate woman of 30, said to have suffered at one time from laryngeal tubercle, with a feebly-united fracture of the femur presenting one and three quarters of an inch of shortening; much disability remaining three months after the injury.

[1] Greiffenhagen, *Deutsche Ztschr. f. Chir.*, 1913, cxxiv. 137. *Ueber den Wert der Hornbolzung und deren Technik.*

[2] Albee, "The Inlay Bone Graft as a Treatment of Ununited Fractures," *American Journal of Surgery*, Jan., 1914.

The fracture, situated just below the middle of the thigh, was exposed by an external longitudinal incision and the feeble lateral union divided, the bone ends were refreshed and replaced in their normal position by Lambotte's traction apparatus, after preparation of the medulla for the peg. A two-inch peg, half an inch in thickness, having been drilled at its centre, was cut from the tibia of the same side just below the tubercle, this was rapidly shaped with bone forceps to fit the medullary cavities, the drill hole having been threaded with stout Pagenstecher thread, the peg was introduced in its whole length into the medullary cavity of the lower fragment and pulled up into the upper cavity to its centre point after Mr. Groves's method. Fixation of the fragments was absolute, a quarter of an inch of shortening only remaining.

The periosteum and muscle was sutured with catgut and the skin with silk gut, all the latter sutures being passed from within out.

The limb was then fixed in a splint, devised by my house surgeon, Dr. Goodden, consisting of a zinc trough with foot-piece, and having a removable zinc cover overlying the femur.

A superficial suppuration occurred at one spot, causing slight temperature, but the wound had soundly healed in three weeks.

The after course was far less satisfactory. At first callus appeared to be forming, but after eight weeks there was no union. Percussion by a rubber hammer after Robert Jones's method was then employed, and although callus appeared, the femur remained ununited.

At the site from which the peg was removed callus also failed to appear, and in a cautious attempt to free some adhesions in the knee-joint the tibia cracked sub-periosteally.

This condition continued for six months, despite all attempts to obtain union. I then removed part of the bone peg and plated. The condition of the peg was of interest. The portion projecting into the upper fragment moved freely in the medullary cavity, and on its being removed by sawing through at the site of fracture, was found to be markedly thinned by deep smooth pits in the compact tissue, and was eburnated at its extremity, but apparently still living. The part of the peg in the lower fragment was vascular, firmly united to the neighbouring bone, and its compact portion almost completely converted into cancellous tissue, showing one's surmise that it might be dealt with as internal callus to be correct.

Following removal of the peg and plating, union took place normally both in the femur and tibia, the patient being discharged with firm union but with limited movement in the knee-joint.

The second case, in a powerful sailor, was identical as regards shortening, but union was firm. A similar proceeding was carried out with a peg two and a half inches in length, but fixation was not so good; the upper fragment having split longitudinally during separation of the bone ends; half an inch of shortening remained.

In this case the limb was put up in a similar manner, but three days later the original shortening was found to be present, the lower fragment with the peg acting as a wedge having been pulled by the powerful muscles into the upper medullary cavity owing to the longitudinal splitting of the compact bone. Extension was, therefore, applied and overdone, for although the alignment of the limb was good, and a quarter of an inch of shortening only remained, the upper end of the peg was shown by skiagram to have been pulled out of the medullary cavity, the compact bone of one side resting on that of the opposite side in the other fragment. A somewhat deeper suppuration occurred, and a sinus remained for some months, eventually closing, but union in the femur was normal as regards time.

This patient also had limited movement in the knee-joint from adhesions, and on being discharged celebrated this by a drunken bout and fell, fracturing his tibia also sub-periosteally at the site from which the peg was removed. This united rapidly, but now seven months after operative treatment the patient is still using sticks to get about, and has considerable disability from muscular atrophy and stiffness of the knee.

The next case was in a well-built man of thirty, who had fractured his right tibia six inches below the knee-joint. One and three quarters of an inch of shortening was present, and despite various attempts, extending over three weeks, to obtain accurate apposition and alignment, the fragments remained in bad position. A peg two and a half inches in length was taken from the crest of the tibia at the site of fracture and introduced in a similar manner to the above. Good alignment and practically no shortening was obtained. Here an aseptic course with primary union obtained. Owing to an improved aseptic technique callus rapidly appeared, but there was some delay in union. Excessive callus eventually appearing, firm union occurred, the patient resuming his ordinary work in fourteen weeks from the time of operation without any discomfort.

The last case, in a delicate man of twenty-five, was a fracture of the left tibia five inches below the joint, but identical as regards shortening and difficulty in reduction. A similar procedure was carried out and primary union occurred. Here again union was markedly delayed and callus very slow in appearing, but three months after the accident the patient could walk on the leg in a Croft splint without discomfort. Later he discarded the splint, and although movement was still present, could walk eight miles without stick or discomfort. Now, six months after the operation very slight movement is still present, but he cycles and walks without pain, although a limp is noticeable, despite the fact that shortening is practically *nil*.

Can one arrive at any conclusions from these few cases? The method in its present form can certainly be said to be unsatisfactory, it is no better than other methods of intra-medullary pegging and inferior to plating, causing both delay in union and irritation. It is possible that the utilisation of bone containing less compact tissue for the peg, such as the fibula, with which medullary cancellous tissue would be placed in continuity with its like, might cause more rapid vascularisation and more rapid absorption or conversion into cancellous tissue, with, as a result, less delay in union and less interference with callus formation in excess or otherwise, and without risk of fracture of the tibia. It is possible that multiple drill holes in the peg might lead to its more rapid absorption, but one fears that this would impair its vitality; and the same may be said of drilling the peg centrally, splitting it longitudinally into layers, and re-uniting it by kangaroo tendon before introduction into the medullary cavity.

The first case certainly proves that bone may retain its vitality, that it may be fairly rapidly absorbed, and that it may be converted into cancellous tissue, being, apparently, dealt with as internal callus at the site of fracture. That mild sepsis reaching the site of fracture is not an unfavourable factor in the formation of callus is shown by the second case, in which union was the most rapid of the series. The sepsis in the first case was scarcely deep-seated enough to affect callus formation in either direction. Destruction of the medulla and consequent interference with local blood supply is not the important factor in non-union, as Cases 2 and 3 united firmly without much delay. The fibula of the same or opposite side should be utilised as more suitable, thus avoiding the accidents that occured in Cases 1 and 2. The presence of the peg as a foreign body is not the only factor in non-union, as Cases 2 and 3 show, and the reason why callus is deficient in one case and in excess in another is yet to seek; with an explanation of this may come the solution of the difficulty.

Since going to press an article has appeared in the *Annals of Surgery*, of April, 1914, by Robinson,[1] recording five successful cases of intra-medullary bone pegging of fractured tibiæ, the peg being obtained from the tibia of the opposite side.

In conclusion, I should like to thank Mr. James Taylor for the many skiagrams taken and for the preparation of lantern slides.

[1] Robinson, "The Treatment of Ununited Fractures of the Tibia by by Bone Transplants," *Ann. Surg.*, April, 1914, p. 495.

